# Canine Smell Preferences—Do Dogs Have Their Favorite Scents?

**DOI:** 10.3390/ani12121488

**Published:** 2022-06-08

**Authors:** Agata Kokocińska, Martyna Woszczyło, Silvestre Sampino, Michał Dzięcioł, Mikołaj Zybała, Anna Szczuka, Julita Korczyńska, Iwona Rozempolska-Rucińska

**Affiliations:** 1 Institute of Biological Bases of Animal Production, University of Life Sciences in Lublin, 13 Akademicka St., 20-950 Lublin, Poland; agata.kokocinska@up.lublin.pl (A.K.); iwona.rucinska@up.lublin.pl (I.R.-R.); 2 Department of Reproduction and Clinic of Farm Animals, Wroclaw University of Environmental and Life Sciences, Grunwaldzki Sq. 49, 50-366 Wrocław, Poland; martyna.woszczylo@upwr.edu.pl; 3 Department of Experimental Embryology, Institute of Genetics and Animal Biotechnology of the Polish Academy of Sciences, Postepu 36A St., Jastrzebiec, 05-552 Magdalenka, Poland; s.sampino@igbzpan.pl; 4 Ethoplanet Ethological Centre of Training and Consulting, Owocowy Sad 53/3 St., 05-500 Piaseczno, Poland; mzybala@ethoplanet.com; 5 Laboratory of Ethology, Department of Neurophysiology, Nencki Institute of Experimental Biology, Pasteur St. 3, 02-093 Warsaw, Poland; a.szczuka@nencki.gov.pl (A.S.); j.korczynska@nencki.edu.pl (J.K.)

**Keywords:** dog, olfaction, smell preferences, cosmetics repellents

## Abstract

**Simple Summary:**

There are many products that are targeted to pet owners. One category of these products is dog repellents—strongly aromatized solutions designed to stop dogs from approaching and investigating particular areas; the second are cosmetics which should be pleasant for dogs. Dogs have a particularly sensitive sense of smell; therefore, strong scents may be very intense, and not always pleasant, stimuli. It is truly interesting, then, that canine cosmetic products often have very strong fragrances designed mostly to appeal to the dog owners, rather than to the dogs themselves. Indeed, the scents that dogs choose to put on their fur differ strongly from those of common cosmetics. Dogs choose mostly intense, animal-derived smells, such as feces or carcasses, so there is a need to differentiate between canine and human smell preferences. As there is limited scientific data related to canine smell preferences, the purpose of this study was to verify dogs’ reactions to selected scents, which can also be appealing to humans. Our study shows that dogs were more likely to interact with the scents of blueberry, blackberry, mint, rose, lavender, and linalol.

**Abstract:**

The available evidence on dogs’ scent preferences is quite limited. The purpose of this study was to verify the canine response to selected odors that may also be preferred by humans. The experiment was performed using 14 adult dogs (10 female and 4 male) of different breeds, body size, and age (1–14 years). During the experiment, dogs were exposed to 33 odor samples: a neutral sample containing pure dipropylene glycol (control) and 32 samples containing dipropylene glycol and fragrance oils. The dog was brought to the experimental area by its handler, who then stopped at the entrance, unleashed the dog, and remained in the starting position. The dog freely explored the area for 30 s. All dog movements and behavior were recorded and analyzed. The methodology of observing the dogs freely exploring the experimental area allowed us to determine the smells that were the most attractive to them (food, beaver clothing). Our study shows that dogs interacted more frequently with the scents of blueberries, blackberries, mint, rose, lavender, and linalol.

## 1. Introduction

Since smell plays such an important role in the life of dogs, gaining an insight into their perception of olfactory communication is an important step in understanding how they experience the world. The perception of smell in most mammals is directly influenced by the structure of the olfactory organ itself. It is made up of two main parts, the main olfactory system and that responsible for the detection of semiochemicals, the vomeronasal organ (VNO), also called the Jacobson organ, which each have separate access routes for fragrances [1,2,3] and communicate with other centers within the central nervous system [4]. In consequence, signals originating from the main olfactory system and the VNO are distinct [5]. This phenomenon allows the detection and recognition of more fragrances than could be predicted on the basis of the number of olfactory receptors alone [6].

In dogs, air is directed to the nose in two separate streams—the respiratory stream and the olfactory stream. During inhalation, the air travels through the top of nasal cavity, and odor particles are deposited on the porous bone structure. While exhaling, the air moves through the lower part of the nasal cavity without disturbing the previously deposited fragrance particles [7]. The epithelium within the main olfactory organ can be divided into two types: the respiratory epithelium and the olfactory epithelium. The main function of the respiratory epithelium is to heat, purify, and moisturize the air entering the nasal cavity, which is facilitated by a layer of mucus on the surface of the epithelium and by ciliated cells [8]. The olfactory epithelium is darkly pigmented and is located on the upper surface of the upper and middle turbinate and in the nasal septum. It is composed of bipolar olfactory cells and support cells that enable it to perform olfactory functions [1,9].

Taking into account the role of olfaction in the canine world, it is likely that dogs could experience a much higher level of exposure to odorants which could be recognized as unpleasant, due to sharing a house environment with their owners. Few available reports suggest, however, that taking into account canine smell preferences could be an important element of enriching and improving the environment shared by the dogs with humans [10]. Moreover, there are data available proving the fact that animals can not only detect and recognize the odor, but also some preferences can be observed based on the animal’s previous experience [11].

Even though the human sense of smell is much less sensitive, and olfaction seems to play a much lesser role in our lives compared to that of animals, and particularly dogs, there is a surprisingly high number of publications dedicated to smell preferences in humans, compared to the extremely low number of similar publications dedicated to dogs [12,13,14,15,16,17,18,19,20,21]. Further, in other species, such as mice, in which olfaction plays a crucial role in many aspects of life, there are a number of publications related to olfaction, including smell preferences [22,23].

It seems very important to think about smells which, in normal life, are imposed upon a dog’s closest environment. Attractiveness of these smells, as well as individual and general dog preferences regarding scents in, for example, animal cosmetics, could have a huge impact on an animal’s everyday welfare and condition.

Smell preference is choosing between different ways to meet the same need. It is based on an ability to evaluate sets of simultaneously available alternatives that satisfy the same motivation and to gravitate towards the most desirable option. A preference may be specific to, and refer to, the difference in motivational strength to get one resource over another, or others. Thus, preferences have a direct impact on an animal’s choices, and thus on its actions. The choice between one resource and another is therefore a decision made on the basis of an analysis of both motivation and preferences. There can be many factors influencing the final choice made by an animal, which is why determining the unequivocal preferences of animals in scientific research can be a challenge.

In laboratory testing on factors that can influence behavior, treatment animals that are exposed to the factor or factors of interest, are contrasted to reference observations. In some experiments, the reference observations are made initially to determine the baseline behavior of each animal. After treatment, the animal is compared to its own baseline as a reference or internal control. In other experimental designs, separate subsets of individuals form a reference or control group that parallels the treatment in all aspects other than the factor(s) of interest. Subsequent comparisons of the control and treatment groups allow scientists to make conclusions about the influences on behavior, with fewer disturbances from intrinsic and extrinsic elements that can confound interpretation [24].

Taking into account the meaning of dogs’ sense of smell as well as the importance of improving animal welfare, which seems in some contexts to be completely dependent upon human choice regarding some environmental factors, we decided to investigate the issue of evaluation of the canine smell preferences in the context of volatile compounds potentially used in the production of pet cosmetics.

## 2. Materials and Methods

### 2.1. Ethical Statement

The research was conducted in accordance with the regulations on animal experimentation and guidelines for the use of animals in research. According to the country’s statute law on animal experimentation, the procedures involving observations of natural behaviors of dogs toward odor or conventional dog training are not animal experimentation; the Local Ethical Commission for Animal Experimentation (resolution no. 67/2014) stated that no special permission for the use of dogs in such noninvasive studies was required.

### 2.2. Animals

The experiment was carried out on 14 adult dogs (10 females and 4 males) of different breeds, body sizes, and ages (Table 1). The dogs’ ages ranged from 1 to 14 years. Three of the males and eight of the females had been neutered. All dogs were pets living in households. Dogs were taken 1 h before the beginning of the test and allowed to run freely in the garden. Each dog was enrolled with its owner and the operator and introduced into the testing arena.

### 2.3. Odor Samples

During the experiment, 33 odor samples were presented to the dogs: a neutral sample containing pure dipropylene glycol (control) and 32 samples containing dipropylene glycol and fragrance oils (Table 2).

Dipropylene glycol is a raw material with low toxicity, making it a popular additive to perfumes, skin and hair care products, and as a liquid used in e-cigarettes. As it is also the most commonly used solvent in dog shampoos, it was used in this study as a control and as a base for diluting fragrance oils. Dipropylene glycol has low toxicity when taken orally, through the skin, or by inhalation. It does not irritate the skin or eyes and there is no evidence that it causes allergic skin reactions. Dipropylene glycol is not considered carcinogenic or genotoxic and has no effect on fertility and reproduction (https://chem-distribution.nl/pl/services/glikol-dwupropylenowy-dpg/ (accessed on 1 February 2022).

Fragrance samples were selected based on fragrances currently used in shampoos and repellents, and smells that might be potentially attractive to dogs, such as fruits that dogs eat. There was no confirmation in the literature about the deterrent effect of specific fragrance oils. There are reports of the purported deterrent effect of citrus, but as they are rather speculative, this was also evaluated in this study.

The odor samples were provided by Pollena Aroma Sp. z o.o. (Nowy Dwór Mazowiecki, Poland). All fragrance oils were diluted in unscented glycol (1:200), which is the most common solvent used in cosmetics.

### 2.4. Experimental Design

**Figure 1:**
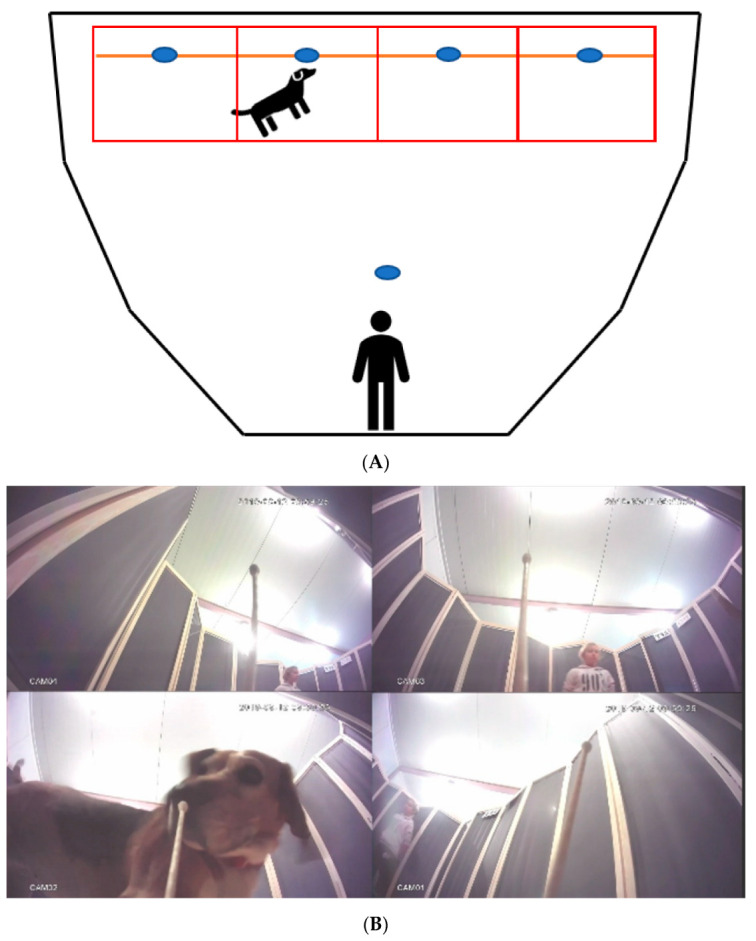
(**A**) Arrangement of the experimental area. The blue dots show the camera positions. The red squares represent zones. (**B**) The view from sample cameras.

Figure 1 shows the arrangement of the area in which the experiment was conducted. The experimental area (size 2.4 m × 3.6 m) was separated from the rest of the room by opaque panels (1.8 m high). The 4 samples were placed in a line near the wall opposite to the entrance, 1 m from one another. Swabs were loaded by immersion into the odor solution, so that much less than 1 mL was presented to the dog. Odor samples were presented on cotton swabs mounted on upright sticks 20 cm high.

When the dog handler entered the experimental area with the dog, they stopped near the entrance, unleashed the dog, and remained at the start position. The dog walked freely in the experimental area for 30 s. All dog movements and behaviors were recorded by 5 cameras, 1 at each sample point and 1 for an overall view. After each trial, the dog was removed from the experimental area and allowed to walk freely outside in the run, before starting the next trial. The experimental area was ventilated and washed twice using clean water before the next dog was introduced.

The double-blind method was used in the research. The sample layout was the same for each dog. Over 8 days, every dog undertook 40 trials (5 trials day). On each day 20 odors were presented to each dog a total of 4 times (Supplementary Table S1). The selection of odors presented in each trial was randomized using Research Randomizer (https://www.randomizer.org (accessed on March 2020). The set of 4 odors presented in each trial was the same for all dogs. In the first trial, only the odor of food was included in the 4 spots, to enhance the dogs’ interest in the odor area.

### 2.5. Behavioral Analyses

Each trial was recorded from the perspective of 5 cameras—1 camera located above the arena in front of the experimenter that covered the entire test area and 4 cameras were located directly next to the samples, so that the animal’s mouth was visible during interaction with the sample (Figure 1B). The recordings were analyzed using the software Observer XT (Noldus Information Technology, Wageningen, The Netherlands). The dog’s interest toward specific odors was explored by analyzing the position of the dog’s head with respect to the owner and the 4 different odor spots.

During the observation, in the zone (1–5, see Figure 1) the animal occupation (involvement) was assessed based on the position of the head. To avoid bias, the person who analyzed the video records of the dogs’ behaviors was blind to the particular samples included in trials. Initially (Study 1), we analyzed the time spent by the dog with its head in the handler zone versus the odor zones. Next (Study 2), we analyzed the proportion of time spent by all dogs in each of the four odor zones in each trial. Then (Study 3), we investigated the preference of the dogs toward specific odors compared to the negative control glycol, by analyzing the proportion of events in all trials in which a specific odor was contacted or ignored in respect to the control. Finally (Study 4), we analyzed the tendency of each dog to use the left, the right, or the left + right nostrils when approaching a specific odor-carrying swab. The full dog ethogram is included in Supplementary Table S2. Moreover, behaviors such as licking the sample, wagging, jumping, yawing, sneezing, wallow, shaking off, and lip licking were taken into account; however, due to the very sporadic nature, the following behaviors were not included in the statistics—no correlation of these behaviors with any particular odor was found.

### 2.6. Statistical Methods

Statistical analyses were conducted using GraphPad Prism, or in R using lme4 package. In Analysis 1, differences in the percentage of time spent with the handler versus the odors’ zones in all trials were analyzed by 2-way ANOVA for repeated measurements, to investigate the influence of the trial versus handler/odors factors on the percentage outcome variable; followed by Bonferroni post-hoc analyses to analyze the differences in each individual trial. In Analysis 2, the percentage of time spent with each of the four odors by the dogs was analyzed using a 1-way ANOVA followed by the Bonferroni multiple comparisons test. Data from both study 1 and study 2 were normally distributed; thus, meeting criteria for ANOVA analyses. In Analysis 3, a generalized linear mixed model was fitted to investigate the association between dog sniffing activity (outcome) and odors (covariates). Sniffing activity of dogs was dichotomized by considering activities lasting more than two seconds as positive interactions, while absence or short-lived interactions were considered null. Similarly, a linear mixer model was used to test the association between duration of sniffing and odors. In both models, glycol was used as the reference category, and the dog IDs were introduced in the model as random effect. Odds ratios (OR) with corresponding 95% confidence intervals (CI) were calculated.

In Analysis 4, the probability of using the right, or left, or right + left nostrils were evaluated for each odor using a binomial logit model to investigate the association between the nostril use (outcome) and odors (covariates).

## 3. Results

### 3.1. Analysis 1

**Figure 2:**
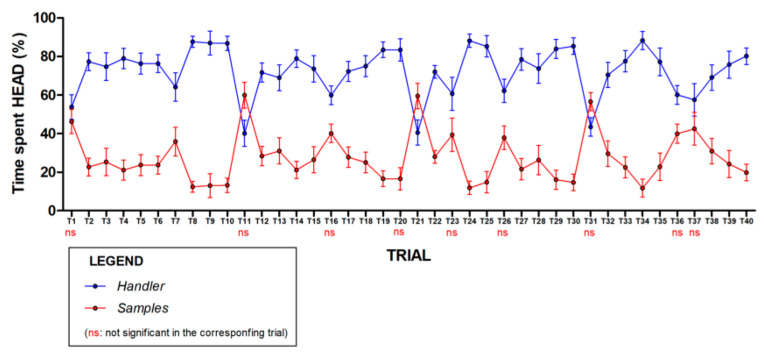

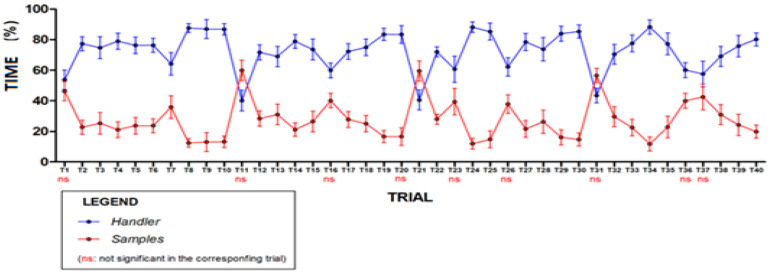
Percentage of time spent by the dog with its head within handler vs. sample areas. Percentage of time spent by the dog with its head within the handler vs. the samples area in each trial. Differences were statistically significant in all trials, except for trials 1, 11, 16, 20, 23, 26, 31, 36, and 37, in which the difference between time spent with handler vs. time spent with samples was not significant.

In this part of the experiment, where the time spent by the dog with its head in the handler zone versus the odor zones was examined, the two-way ANOVA for repeated measures showed a significant effect of the handler (F = 194.73_(1,16)_, *p* < 0.0001) on the dog position over the trial factor, which was not significant. Bonferroni post-hoc analyses showed that, in most trials, the dogs spent significantly more time in the handler zone, with relatively little time exploring the odor swabs. However, in trials 1, 11, 16, 21, 23, 26, 31, 36, and 37, the difference in the time spent between handler versus odor zones was not significant (Figure 2), indicating that the dogs spent more time in the smell zones. Interestingly, among the odors presented in these specific trials a positive control (food and castor odors), was always present. This result indicates that when an odor was of interest, the dog spent more time in the odor zones. Moreover, the percentage of time spent with the handler by hunting dogs versus other breeds was analyzed by 2-way ANOVA, which showed no effects of the breed factor (F = 1.37_(1385)_, *p* = 0.24) and a significant effect of the trial on the percentage outcome variable (F = 4.64_(39,85)_, *p* < 0.0001). Similarly, there were no significant effects of the dogs’ sex on the percentage of time spent with the handler or in the odor zones.

### 3.2. Analysis 2

**Figure 3:**
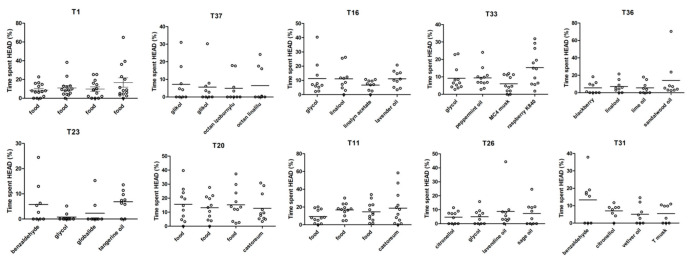
Percentage of time spent by the dog with its head within the samples presented in selected trials. Each dot represents an individual dog. No significant differences were observed in the time spent by the dog among the four smells presented in the single trial.

Analyses of the percentage of time spent by all dogs in each of the four odor zones in the single trials showed that there were little differences between the four smells in each trial (Figure 3). In particular, the odor of food was always preferred by the dogs over the other smells presented in the same trial. In addition, tangerine and raspberry odors were significantly more explored in trials 23 and 33, respectively, compared to other odors presented in the same trial (Figure 3). In these two trials, hunting dogs displayed similar levels of exploration compared to the other breeds.

### 3.3. Analysis 3

The dog position toward a specific odor or handler zone is an indicator of the dog’s interest; however, it does not reveal whether the dog is actively sniffing the odor swabs. Therefore, a more accurate analysis of the dog behavior when approaching the swabs was conducted by direct observation of the dogs’ sniffing activity and its duration. First, all trials were analyzed using a generalized linear mixed model to investigate which odors had a higher probability of being sniffed by the dogs as compared to the control glycol (Table 3). A positive interaction was considered when the duration of sniffing was higher than 2 s. This model revealed that the positive controls, food and castoreum, had the highest probability of being contacted and sniffed, while orange oil had a significantly higher probability of being sniffed compared to glycol, although to a lesser extent with respect to the positive controls. In addition, the linalyl acetate was explored with less frequency as compared to glycol. Such preference trends were true even taking into account the dog ID as a random factor. Furthermore, to investigate which odors had the highest probability of being sniffed for a longer period of time, the dataset was first filtered to remove all interactions in which the duration of sniffing was less than 2 s, and a linear mixed model was run to explore which odors arouse a more durable interaction as compared with the negative control glycol (Table 4). The highest probability of displaying a durable sniffing was found for lime oil and ambrettolide, while other odors (e.g., isobornyl acetate, linalyl acetate, globalide, beta pinene, basil oil, and beta ionone) were still arousing significantly longer interactions as compared with the control glycol, but to a lesser extent. Interestingly, this analysis showed that the positive controls, food and castoreum, were not significantly associated with durable sniffing as compared to glycol.

### 3.4. Analysis 4

The results of analyses of the tendency of the dog to use the left, the right, or the left + right nostrils when approaching a specific odor revealed a higher probability of using the left or the left + right nostrils, and only rarely using the single right nostril, when dogs encountered lavandine oil, even after correcting for individual dogs’ variability as a random effect.

## 4. Discussion

It seems very important to consider the smells which, in normal life, are part of a dog’s closest environment. The attractiveness of these smells, and individual and general dog preferences according to different scents in, for example, animal cosmetics, could have a huge impact on everyday animal welfare and condition.

Many studies point to the early development of olfactory structures and their impact on the interaction of an individual with its environment; thus, at the same time the issue of olfactory preferences may arise as early as in the fetal period [25,26,27,28]. However, research on dogs’ scent preferences is very limited. In the pet industry, the database focuses on methods used to determine food acceptance or preference by pets. Despite a food odor’s attractiveness, some studies have focused on odor preferences in individuals in terms of health or disease in various species [29], phases of the sexual cycle [13,30,31], or even the influence of specific personality traits on olfactory preferences [32,33,34]. It is worth mentioning that body odor which could influence an individual’s perception may change in connection with disease [35,36,37,38], emotional states [39], diet, or hormonal changes [40,41,42].

In studies on the communication and cognitive abilities of dogs in a social context, much more emphasis has been placed on the analysis of visual communication [43,44,45,46,47,48,49,50,51] than olfactory abilities [52]. We do not know exactly how strongly dogs base their orientation in the environment on olfactory cues, but we have a conviction about the enormous possibilities of perceiving a dog’s sense of smell [53].

Animals may be motivated to interact, or to avoid interacting, with a given stimulus. Positive and negative motivations are inferred from behavior, for example, from approach-behavior vs. avoidance, respectively [54]. Individuals of one species may differ in the strength of their motivation for specific stimuli. These differences are explained by many factors, ranging from sex, genes, and age to the developmental environment or the individual’s acquired experience. Motivation is generally influenced by many factors that can be intrinsic (e.g., genetic or physiological) or extrinsic (i.e., in the animal’s environment), for example, the motivation to drink (thirst) can be increased by hormones responsible for controlling the body’s water balance but is also enhanced by the sight of water [24].

It is difficult to evaluate preference tests in captive or wild-born animals because it is not certain that the same animal would still have the same choice without such significant environmental changes. This is a very common problem when it comes to choosing between several resources for laboratory testing. In our study, this fact should have been also taken into consideration—the fact that an animal selects a given smell under laboratory conditions is not yet a scientific confirmation that under natural conditions it would still choose that smell. Although the results of our study indicated that dogs spent more time near samples of edible plants (such as berries), it should be noted that the choice of the scent itself does not have to be related to the fact that the animal would like to have such a scent on its fur. However, while fragrance preference testing, especially in relation to animal cosmetics, is still a nascent research field, it should be remembered that forcing an animal to wear a scent that it finds to be less unpleasant may have a less negative impact on its welfare, than forcing it to wear a scent that animals avoid, even under laboratory conditions.

We tried to choose a methodology that would be as natural as possible for observation of free exploration, without human influence, while still in the presence of a handler, so that the dog felt safe. A double-blind test was applied: the dog and handler did not know what kind of odor samples were used in each trial. Preference laboratory testing is usually based on presenting the animal with several possible stimuli and determining the frequency of the choice made. In our research, as well as latency, inhalation time, and sample selection frequency, we also analyzed other behaviors that are adopted as affiliate behaviors (related to the pursuit of the object, interest, feeling of pleasure, behaviors related to a detailed analysis of smell, such as: tasting, rubbing, drooling, licking), and stressful behavior (avoidance reaction, sneezing). Next, when the animal’s choice behavior reveals the option that is preferred or avoided in the experiment, the scientist can move on to determine the strength of its behavioral preference. Such tests are termed preference tests.

Our results showing increased time spent by dogs in the zone of some odors confirmed that some of them aroused more interest than others. Moreover, in the case of lavender, increased proportions of using left and left + right nostrils could suggest that this odor could be recognized by the dogs not only as interesting but also pleasant [55,56]. Apart from the food odor, some other, less-obvious odors such as blueberry, peppermint, castor, tangerine, and rose were found to be as interesting. The interest of animals, in particular plants, can be somehow explained by its usefulness, for example, in the process of self-curing, or protecting against parasites. There are dozens of examples of that kind of practice described in many species (mammals, reptiles, and birds); however, no examples are known to us regarding that kind of behavior observed in domestic dogs. That can be explained by the limited possibilities of presenting that kind of behavior in this species [57]. Our study shows that the smell of food and the smell of beaver clothing are attractive to dogs. The animals explored the positive controls with more frequency with these samples (89.19% contact time for beaver clothing and 87.44% with food samples), also licking and the dogs’ clear interest in these samples being noted (sample licking only happened with positive controls, however, only a few dogs performed this behavior sporadically, which is not statistically significant). The other smells in the study can thus be compared to interactions with the control samples.

We found that dogs were more likely to interact with the scent of lavender, so it can be assumed that this smell is not unpleasant or repellant for them. Lavender has been proven to affect a variety of species (including dogs and humans), with the scent of lavender shown to lower the heart rate of dogs, possibly by affecting vagal activity, as well as increasing the rest and sitting time while riding in a car [58,59]. Similar results were also obtained in a shelter study—dogs exposed to the scent of lavender were calmer. Similar results were achieved with the chamomile fragrance. Conversely, peppermint and rosemary had stimulating effects on dogs, who tended to be more agitated and spend less time resting when exposed to these fragrances [10].

The scent of lavender can also affect human’s emotional state, reducing the effects of oxidative stress and lowering cortisol levels [60]. Smelling lavender and rosemary increases free radical scavenging activity and decreases cortisol level in saliva [61].

The sense of smell in dogs is not only a highly developed sense, but also plays a huge role in the animal’s welfare. The smell can also be combined with the individual preferences of the animal [62], which in turn can be modulated by previous experiences. The ability of odors to evoke past memories has been shown in humans, as well as in dogs [63,64,65]. The ability of odors to evoke emotional memories has been demonstrated in people suffering from posttraumatic stress disorder (PTSD). It is still an open question if a similar mechanism is present in dogs in the context of pleasure and unpleasure memories. These examples show that fragrance selection must be performed carefully, and that commercially available dog cosmetic products should be varied in composition so that the owner (and, indirectly, their dog) has a choice.

Although the particular scents evaluated in our study, and especially those found to be more interesting for dogs, were supposed to be neutral to dogs in the context of their previous experience, it seems worthwhile mentioning that proper (gentle and stressless) introduction might be a very important issue, since stress generated during the first exposure to the scent (e.g., first bath) can create a negative association, which would destroy the positive reception to the scent postulated in this study. The association of a particular scent with some emotions can influence the perception of the scent.

Another issue that could be discussed in the context of the use of cosmetics in dogs, apart from attractiveness, is its influence on self-recognition. In the Horowitz study related to this issue, the authors concluded that dogs show more investigative interest in their own odors when modified [66]. As mentioned previously, animals have a natural tendency to modify their own scent, which could be used as a natural scent camouflage, but also could be caused by the desire to obtain a repellent effect [67].

The effect of a smell on a pet’s behavior is not the only way to determine its possible preferences or to distinguish between the positive or negative effects of a particular odor on a dog. The intense sniffing of the sample with licking, observed in our study in the case of food and beaver suit samples, can also be considered an indicator of increased interest and positive associations the animal has with the odor of the sample. Similar behaviors occurred in interaction with other odors such as lavender, rose, and blueberries.

Smell can also be treated as an enrichment of the environment, encouraging even more frequent interactions with toys by dogs in a shelter [68]. Enrichment of the environment is one of the key tools in improving the welfare of captive animals, although to meet this objective, the enrichment must be important and pleasant for the animal. Since odors have been shown to affect human mood [20], it can be also taken into consideration that dogs, for whom the sense of smell is much more important than for the human, can also be influenced in that way.

Since we noticed that among others the rose scent appeared to be interesting (and possibly pleasant) to the dogs, we were curious about the biological sense and the practical explanation for that kind of preference. However, in this context it is worth mentioning that the same or similar fragrances can have very different origins [69]. In many cases, animals, including mammals, significantly contribute to pollination by serving as vectors for pollen transfer [70]. Moreover, the scent recognized as a rose scent, taking into account that it usually has more than one component, could be sensed much differently by dogs, which could detect another component as dominant [70].

The experimental setup included two types of positive samples (food-sausage and castoreum) in order to validate the experiment. Both were scents of a certain attractiveness and turned out to be the most preferred odors by dogs, which confirms the correctness of the experimental design.

Other fragrances that turned out to be highly attractive to dogs and their selection and were statistically significantly more frequent than that of other samples were: 1. Peppermint; 2. Rose; and 3. Blueberry.

There is no scientific research about dog preferences for these smell, but we can find reports about wolves’ reactions to some fragrances. For example, in the book: “The truth about wolves and dogs” written by Toni Shelbourne [71], we can find the sentence: Strong smells like perfume, fabric conditioner or mint can over-stimulate wolves and if a peppermint is eaten before interaction with them, they will lick around your mouth or bible-groom you on the chin.

However, there is no given source for this statement or scientific basis; therefore, it should be treated as an anecdotal source. The only one scientific report about peppermint was in the experiment in a shelter, where the diffusion of rosemary and peppermint into a dog’s environment encouraged significantly more standing, moving, and vocalizing than other types of odors [10].

However, these reports do not analyze the attractiveness or aversion to this fragrance.

The scent of the mint itself was chosen by the author. She noticed that many of her dogs and client’s dogs eat the mint planted in their home gardens.

The logical dog’s choice was the aromas of blueberry and wild rose (*Rosa canina* L.) (also known as the dog rose). The author, choosing these fragrances, was guided by reports that wolves eat the fruits of these plants to supplement their diet [72,73]. Dogs are also happy to heal themselves by eating these fruits. The fruits of dog rose are a very popular component of the BARF diet, and we can buy a lot of supplements for dogs with these fruits (e.g., Game dog BARFER Rose Hip). Our research confirmed the attractiveness of their scent.

There were also some smells totally neutral for dogs or that dogs avoid with statistical significance, such as linalyl acetate. An interesting fact is that citronellol has for humans a rose smell, and geranium has a rose-mint smell, globalide is an imitation of the musk smell. However, the human nose is much easier to deceive than a dog’s sense of smell.

## 5. Conclusions

Our study shows that dogs were more likely to interact with the scents of blueberry, blackberry, mint, rose, lavender, and linalol so it can be assumed that these smells were not unpleasant or avoidable for them. The methodology of observing dogs freely exploring research sites allows the determination of the smells that are most attractive to them (food, beaver clothing). Further study seems to be required in the context of the importance of the intensity of the scents used in cosmetics production (differences in the scent detection thresholds of humans and dogs) and the duration of persistence of the scents on the animals’ fur. The prevalence of sex-dependent preferences regarding particular scents might also be worthy of investigation.

## Figures and Tables

**Table 1:** Dogs used in experiment.

Dog	Breed	Age (Years)	Sex	Neutered
D1	Polish Hunting Spaniel	1	F	+
D2	Polish Hunting Spaniel	1	M	+
D3	Polish Hunting Spaniel	6	F	+
D4	Polish Hunting Spaniel	3	F	+
D5	Mixed	2	M	+
D6	Mixed	8	F	+
D7	Bearded Collie	9	M	−
D8	Tibetan Terrier	14	M	+
D9	Shih Tzu	10	F	−
D10	Polish Hunting Spaniel	5	F	−
D11	Beagle	8	F	+
D12	Beagle	8	F	+
D12	Beagle	9	F	+
D14	Beagle	10	F	+

**Table 2:** Odor samples used in the experiment.

Sample No.	Odor Sample
1	peppermint oil
2	blackberry K756
3	isobornyl acetate
4	vetiver oil
5	linalool
6	citronellol
7	sage oil
8	musk MC4
9	linalyl acetate
10	melon C186
11	tangerine oil
12	benzaldehyde
13	globalide
14	rosewood oil
15	sandalwood oil
16	orange oil
17	rose oil
18	lime oil
19	beta pinene
20	lavandine oil
21	strawberry K814
22	Eugenol
23	lavender oil
24	basil oil
25	raspberry K840
26	ambrettolide
27	beta ionone
28	blueberry D761/G
29	geranium oil AT018
30	T musk
31	glycol (negative control)
32	food—meat (positive control)
33	castoreum (positive control)

**Table 3:** Dogs’ positive interaction—sniffing activity (Yes/No): generalized linear mixed model displaying associations between sniffing activity (outcome) and administered odors (covariates), with dogs’ IDs as a random factor and glycol as the reference category.

	Dogs’ Positive Interaction		
*Predictors*	*Odds Ratios*	*CI*	*p*
(Intercept)	0.58	0.39–0.86	0.007
Peppermint oil	1.72	0.90–3.27	0.098
Blackberry K756	0.91	0.47–1.77	0.779
Isobornyl acetate	1.00	0.52–1.93	1.000
Vetiver oil	0.62	0.31–1.26	0.188
Linalool	1.00	0.52–1.93	1.000
Citronnellol	1.00	0.52–1.93	1.000
Sage oil	1.44	0.76–2.74	0.267
Musk MC4	0.80	0.41–1.56	0.511
Linalyl acetate	0.42	0.20–0.90	0.026
Melon C186	0.82	0.39–1.76	0.620
Tangerine oil	0.89	0.49–1.64	0.712
Benzaldehyde	0.67	0.34–1.34	0.261
Globalide	0.54	0.26–1.11	0.092
Rosewood oil	0.91	0.47–1.77	0.779
Sandalwood oil	0.75	0.38–1.47	0.399
Orange oil	2.46	1.28–4.70	0.007
Rose oil	1.57	0.83–2.99	0.167
Lime oil	0.67	0.34–1.34	0.261
Beta pinene	0.82	0.42–1.62	0.575
Lavandine oil	0.75	0.38–1.47	0.399
Strawberry K814	1.57	0.83–2.99	0.167
Eugenol	0.82	0.42–1.62	0.575
Lavender oil	1.10	0.57–2.11	0.780
Basil oil	0.91	0.47–1.77	0.779
Raspberry K840	1.53	0.74–3.16	0.252
Ambrettolide	1.20	0.67–2.18	0.541
Beta ionone	0.91	0.47–1.77	0.779
Blueberry D761/G	0.94	0.44–1.98	0.869
Geranium oil AT018	0.54	0.26–1.11	0.092
T musk	0.96	0.53–1.76	0.902
Food-meat	3.45	2.31–5.16	<0.001
Castoreum	6.17	2.63–14.47	<0.001
**Random Effects**			
σ^2^	3.29		
τ_00 Dog_	0.26		
ICC	0.07		
N _Dog_	12		
Observations	1920		
Marginal R^2^/Conditional R^2^	0.082/0.149		

**Table 4:** Sniffing activity (duration): linear mixed model displaying associations between duration of sniffing activity (outcome) and administered odors (covariates), with dogs’ IDs as a random factor and glycol as the reference category.

	Time		
*Predictors*	*Estimates*	*CI*	*p*
(Intercept)	3.05	2.42–3.69	<0.001
Peppermint oil	−0.42	−1.51–0.67	0.452
Blackberry K756	0.14	−1.23–1.50	0.843
Isobornyl acetate	1.55	0.26–2.84	0.019
Vetiver oil	0.51	−0.75–1.76	0.428
Linalool	0.59	−0.74–1.92	0.385
Citronnellol	0.52	−0.59–1.63	0.362
Sage oil	0.23	−1.06–1.52	0.724
Musk MC4	0.28	−0.92–1.48	0.646
Linalyl acetate	1.60	0.34–2.85	0.013
Melon C186	0.86	−0.40–2.12	0.180
Tangerine oil	0.44	−0.63–1.52	0.420
Benzaldehyde	0.41	−1.05–1.87	0.585
Globalide	1.30	0.04–2.56	0.043
Rosewood oil	0.80	−0.61–2.22	0.266
Sandalwood oil	0.81	−0.66–2.28	0.278
Orange oil	1.22	−0.01–2.45	0.052
Rose oil	0.69	−0.44–1.82	0.230
Lime oil	1.91	1.26–2.56	<0.001
Beta pinene	1.61	0.57–2.65	0.002
Lavandine oil	0.39	−1.03–1.80	0.593
Strawberry K814	0.72	−0.50–1.95	0.249
Eugenol	−0.40	−1.62–0.83	0.526
Lavender oil	0.58	−0.57–1.73	0.323
Basil oil	1.43	0.30–2.56	0.013
Raspberry K840	0.49	−0.80–1.78	0.460
Ambrettolide	2.88	1.29–4.47	<0.001
Beta ionone	1.49	0.13–2.86	0.032
Blueberry D761/G	−0.01	−1.47–1.45	0.986
Geranium oil AT018	0.84	−0.42–2.10	0.189
T musk	0.86	−0.47–2.18	0.205
Food-meat	0.17	−0.86–1.19	0.750
Castoreum	−0.31	−1.42–0.80	0.581
**Random Effects**			
σ^2^	5.83		
τ_00 Dog_	0.46		
ICC	0.07		
N _Dog_	12		
Observations	775		
Marginal R^2^/Conditional R^2^	0.087/0.154		

## Data Availability

The data that support the findings of this study are available from the corresponding author (A.K.), upon reasonable request.

## Supplementary Materials

The following supporting information can be downloaded at: https://www.mdpi.com/article/10.3390/ani12121488/s1, Table S1. Arrangement of samples. Table S2. Dog ethogram.
