# Supplementary material for: Canine Smell Preferences—Do Dogs Have Their Favorite Scents?
Source: Animals (Basel). 2022 Jun 8;12(12):1488. doi: 10.3390/ani12121488 (PMC9219509; doi:10.3390/ani12121488)
Supplement: Supplementary file 1 [file animals-12-01488-s001.zip › animals-1644336-supplementary.pdf]

Supplementary Table S1. Arrangement of samples.

Trial	Sample position	Day 1	Day 2	Day 3	Day 4	Day 5	Day 6	Day 7	Day 8
1	1	32	12	32	5	33	31	32	25
1	2	32	15	33	31	32	1	32	1
1	3	32	20	32	9	32	2	32	34
1	4	32	16	32	23	32	16	33	8
2	1	27	26	27	3	14	31	11	21
2	2	17	13	25	16	22	6	31	32
2	3	1	28	17	8	26	20	17	23
2	4	21	24	31	31	30	7	14	26
3	1	29	10	28	10	31	23	12	7
3	2	18	4	32	22	32	25	4	28
3	3	32	3	18	14	17	15	6	20
3	4	31	6	31	31	3	10	30	31
4	1	31	25	20	26	13	5	29	18
4	2	2	23	1	30	31	4	22	5
4	3	7	9	7	2	11	8	10	15
4	4	14	11	13	4	12	24	13	2
5	1	19	8	21	19	9	28	27	9
5	2	5	31	29	11	19	18	24	31
5	3	31	22	12	6	31	21	19	31
5	4	31	30	24	15	29	27	16	3

Supplementary Table S2. Dog ethogram.

Behavior Name	Description	Start code	Stop code	Behavior Type
AREAS	Being in sample area (mutually exclusive)			State Event
Handler area		s5		State Event
Sample 1		s1	T	State Event
Sample 2		s2	R	State Event
Sample 3		s3	E	State Event
Sample 4		s4	F	State Event
HEAD	Head of the dog being in sample area (mutually exclusive)			State Event
Handler area		g5	A	State Event
Sample 1		g1	G	State Event
Sample 2		g2	L	State Event
Sample 3		g3	O	State Event
Sample 4		g4	W	State Event
SNIFFING	Sniffing in sample area (mutually exclusive)			State Event
Sample 1		w1	Z	State Event
Sample 2		w2	Y	State Event
Sample 3		w3		State Event
Sample 4		w4		State Event
Handler area		w5	5	State Event
SPECIFIC SNIFFING	Clasification of sniffing type (mutually exclusive)			State Event
not sniffing	Not interested in samples area	kw	N	State Event
upper wind 1	upper wind in sample area	u1	ku1	State Event
upper wind 2	upper wind in sample area	u2	ku2	State Event
upper wind 3	upper wind in sample area	u3	ku3	State Event
upper wind 4	upper wind in sample area	u4	ku4	State Event
NOSTRILS	clear view on which nostril is close to the sample			State Event
1 sample left nostril		nl1	kl1	State Event
1 sample right nostril		np1	kp1	State Event
2 sample left nostril		nl2	kl2	State Event
2 sample right nostril		np2	kp2	State Event
3 sample left nostril		nl3	kl3	State Event
3 sample right nostril		np3	kp3	State Event
4 sample left nostril		nl4	kl4	State Event
4 sample right nostril		np4	kp4	State Event
1 sample whole nose touch		c1	kc1	State Event
2 sample whole nose touch		c2	kc2	State Event
3 sample whole nose touch		c3	kc3	State Event
4 sample whole nose touch		c4	kc4	State Event
LATENCY				State Event
latency 1	Time needed for dog to sniff the sample directly	1	d1	State Event
latency 2	Time needed for dog to sniff the sample directly	2	d2	State Event

latency 3	Time needed for dog to sniff the sample directly	3	d3	State Event
latency 4	Time needed for dog to sniff the sample directly	4	d4	State Event
